# Creation of nano eye-drops and effective drug delivery to the interior of the eye

**DOI:** 10.1038/srep44229

**Published:** 2017-03-14

**Authors:** Yoshikazu Ikuta, Shigenobu Aoyagi, Yuji Tanaka, Kota Sato, Satoshi Inada, Yoshitaka Koseki, Tsunenobu Onodera, Hidetoshi Oikawa, Hitoshi Kasai

**Affiliations:** 1 Institute of Multidisciplinary Research for Advanced Materials, Tohoku University, 2-1-1 Katahira, Aoba-ku, Sendai, Miyagi 980-8577, Japan; 2 Ouchi Shinko Chemical Industrial Co., Ltd., Research and Development Center, 111 Shimojyukumae, Sukagawa, Fukushima 962-0806, Japan; 3 Graduate School of Medicine, Tohoku University, 2-1 Seiryo-machi, Aoba-ku, Sendai, Miyagi 980-8575, Japan

## Abstract

Nano eye-drops are a new type of ophthalmic treatment with increased potency and reduced side effects. Compounds in conventional eye-drops barely penetrate into the eye because the cornea, located at the surface of eye, has a strong barrier function for preventing invasion of hydrophilic or large-sized materials from the outside. In this work, we describe the utility of nano eye-drops utilising brinzolamide, a commercially available glaucoma treatment drug, as a target compound. Fabrication of the nanoparticles of brinzolamide prodrug increases the eye penetration rate and results in high drug efficacy, compared with that of commercially available brinzolamide eye-drops formulated as micro-sized structures. In addition, the resulting nano eye-drops were not toxic to the corneal epithelium after repeated administration for 1 week. The nano eye-drops may have applications as a next-generation ophthalmic treatment.

Drug delivery systems (DDSs) have been widely investigated to improve delivery of physiologically active agents to tumours^1^,^2^,^3^,^4^,^5^,^6^, inflamed areas^7^ and the bowels^8^,^9^. For selective delivery to tumours, for example, polymeric micelles, hydrogels, and liposome have been used as nano-sized carriers (approximately 100 nm in size) to achieve enhanced permeation and retention (EPR) effects^10^. Effective delivery of drugs would allow clinicians to reduce overall drug doses and should result in fewer side effects. We previously reported that the nanoparticles of dimeric compounds of SN-38 or podophyllotoxin (PPT), i.e., water-insoluble anticancer agents, without nano-sized carriers showed enhanced cytotoxicity in cancer cells compared with those of clinically available water-soluble analogues^11^,^12^,^13^,^14^,^15^. These nanoparticles were obtained using our original fabrication technique for nanoparticles (nanocrystals) called the reprecipitation method^16^. The reprecipitation method is a simple method for creating organic nanoparticles based on the differences in solubility between poor solvents and solutions of the target compound. We have fabricated many types of nanocrystals, such as polydiacetylenes^17^,^18^, pigments^19^,^20^, and organometallic compounds^21^, using this method.

The normal corneal layer has several tight barriers, including the hydrophobic epithelium and hydrophilic stroma^22^,^23^,^24^,^25^. These layers generally prevent water-soluble molecules, which are hydrophilic, from entering the eye through the epithelium of the cornea. Hydrophobic microparticles, which are relatively large, are also blocked from entering the eye by the fibrous layer of the stroma, due to their size. This leads us to assume that nanoparticles could easily penetrate the eye through the cornea. These particles are hydrophobic and very small, and should thus be able to pass through both the epithelium and the stroma. Our group has previously observed that fluorescent nanoparticles, fabricated by the reprecipitation method, were indeed able to penetrate the cornea^26^. In addition, studies of DDSs to deposit drugs into the eye using nano-sized carriers, e.g., polymeric micelles, liposomes, and solid lipid nanoparticles, have previously been published^23^,^27^,^28^,^29^,^30^,^31^.

Nanoparticle DDSs are a particularly promising part of improved treatments for glaucoma. This very common ophthalmic disease is characterized by the degeneration of the retinal ganglion cells (RGCs) and irreversible loss of the visual field^32^,^33^. In animal models, RGC death in glaucoma can be induced by various stress including oxidative stress, ER stress and calpain activation after axonal damage^34^,^35^,^36^,^37^,^38^. The only established treatment to prevent RGC loss is lowering intraocular pressure (IOP), typically achieved by topical application of hypotensive medications as eye-drops. In this study, we chose brinzolamide (Fig. 1) as the target compound because it is the major therapeutic component in Azopt, a type of commercially available eye-drops for glaucoma, which is effective for decreasing IOP^39^,^40^,^41^. The concentration of brinzolamide in Azopt is very high (1%) because the brinzolamide suspension liquid in Azopt consists of relatively large, micro-sized structures (more than 10 μm in size; Supplementary Fig. 3). The high concentration of brinzolamide makes Azopt cloudy, often leading to patient discomfort after administration^42^. We speculated that a nanoparticle-based brinzolamide delivery system would have a stronger ocular hypotensive effect at a lower concentration because of more effective drug delivery, as well as higher transparency because of its lower particle size. Thus, this study aimed to develop eye-drops including nanoparticles of a physiologically active agent. These nano eye-drops promise to be a critical part of next-generation ophthalmic treatments.

## Results

### Synthesis of brinzolamide prodrugs

We first attempted to fabricate brinzolamide nanoparticles using the reprecipitation method. However, only micro-sized particles of brinzolamide were obtained (Supplementary Fig. 4). The solubility of the target compound in poor solvents is thought to be closely related to the sizes of particles obtained by the reprecipitation method^43^. Therefore, the solubility in a poor solvent should be decreased in order to fabricate smaller nanoparticles. Accordingly, brinzolamide was modified by different hydrophobic substituents in order to decrease its solubility in water during the reprecipitation.

**Figure 1:**
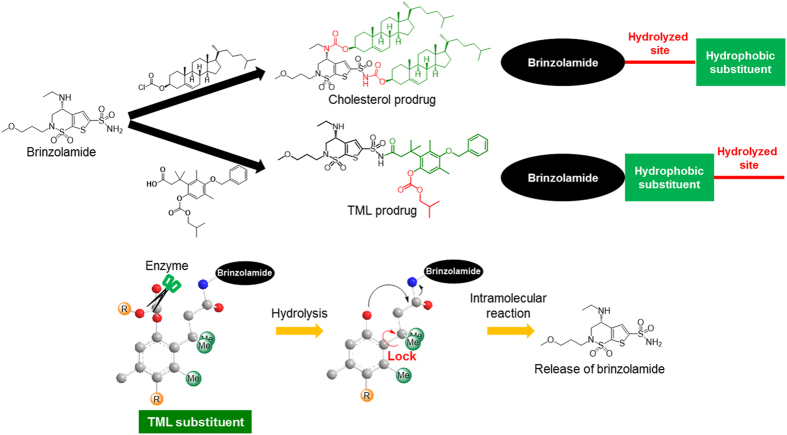
Chemical structures of brinzolamide, cholesterol prodrug, and trimethyl lock (TML) prodrug. The hydrolysed sites of the cholesterol prodrug were located inside molecule, whereas the hydrolysed site of the TML prodrug was located at the end of the molecule. The TML substituent could easily release brinzolamide because the three methyl groups prevented the rotation of the C-C bond. Scissors indicate hydrolase enzyme.

First of all, cholesterol was selected as a hydrophobic substituent because of its high hydrophobicity and low toxicity for human body. Cholesterol prodrug was synthesised by the linking reaction between brinzolamide and two equivalents of cholesterol chloroformate through hydrolysable carbamate bonds (Fig. 1). The chemical structure of the cholesterol prodrug was characterised using IR spectroscopy, ^1^H and ^13^C NMR spectroscopy, and high-resolution mass spectrometry (HRMS). The cholesterol prodrug had to be hydrolysed by enzymes present in the eye in order to release brinzolamide and exert its ocular hypotensive effects^39^.

The prodrug of trimethyl lock (TML), which easily undergoes hydrolysis, was also considered in order to decrease the solubility in water and to realise the release of brinzolamide in the eyes. The TML substituent could easily release brinzolamide because of the existence of the trimethyl locked structure in the hydrophobic substituent^44^. That is, adjacent three methyl groups of TML substituent fix the conformation of C-C bond consist of aromatic ring and the aliphatic sidechain bearing brinzolamide to make close carbonate and amide moieties (Fig. 1). Enzymatic hydrolysis of carbonate to afford phenolic hydroxyl group triggered smooth intramolecular cyclization to form dihydrocoumarine and release brinzolamide.

The TML precursor was afforded through an eight-step reaction (Supplementary Fig. 1)^44^,^45^,^46^. The TML prodrug was then prepared by a condensation reaction between brinzolamide and the TML precursor using EDC and DMAP. In contrast to the cholesterol prodrug, the disubstituted TML prodrug was not obtained under these conditions despite the existence of two amine groups in brinzolamide. Only the TML precursor-monosubstituted brinzolamide was identified by both NMR and MS. The position of the substituted amine group was determined by permethylation of the amine groups within the TML prodrug (Supplementary Fig. 2) and measurement of ^1^H-detected multibond heteronuclear multiple quantum coherence spectrum (HMBC). The correlation between the ^1^H NMR spectra of the methyl group (2.22 ppm) modified in the ethylamine group with the ^13^C NMR spectra of the methylene chain (47.9 ppm) in the ethylamine group and the C4 carbon (55.6 ppm) of the brinzolamide moiety was observed (Supplementary Fig. 5). The results showed that the TML substituent was connected with the sulphonamide group of brinzolamide.

### Fabrication of brinzolamide prodrug nanoparticles

**Figure 2:**
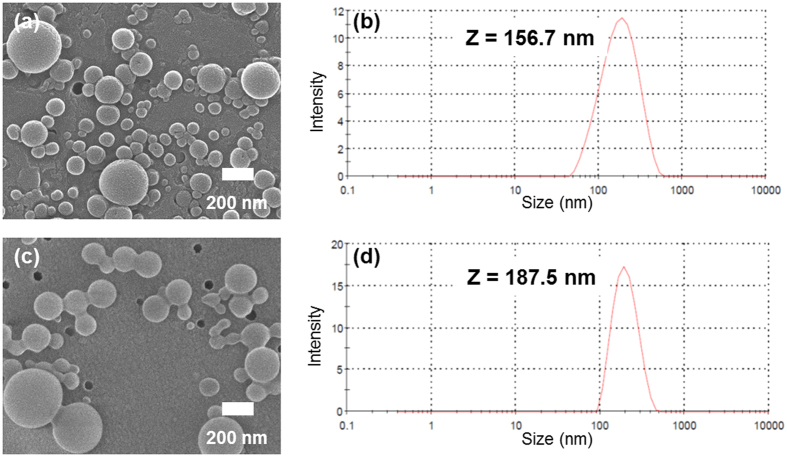
SEM images of cholesterol prodrug nanoparticles (**a**) and TML prodrug nanoparticles (**c**). The size distributions of cholesterol prodrug nanoparticles (**b**) and TML prodrug nanoparticles (**d**) in aqueous media were measured by DLS.

In both cholesterol- and TML-substituted compounds, nanoparticles of brinzolamide prodrugs were obtained by the reprecipitation method. A solution of brinzolamide prodrug in acetone with surfactant was injected into stirring distilled water. After reprecipitation, the aqueous dispersions of brinzolamide prodrug nanoparticles were dialysed to remove organic solvent. Despite the high concentration of brinzolamide prodrug (0.5 wt%), brinzolamide prodrug nanoparticles were clearly dispersed for more than few days because of their high zeta potential (Supplementary Fig. 6) and addition of surfactant. As shown in Fig. 2, nanoparticles of the cholesterol and TML prodrugs (approximately 200 nm in size) were fabricated, observed by scanning electron microscopy (SEM), and measured by dynamic light scattering (DLS).

The light transmittance of the aqueous dispersion of the TML prodrug nanoparticles was measured and compared with Azopt (Supplementary Fig. 7). The aqueous dispersion of the TML prodrug nanoparticles had a maximum transmittance of approximately 20% at a wavelength of around 800 nm. On the other hand, the maximum transmittance of Azopt at this wavelength was 0.2%. Thus, the transmittance of the aqueous dispersion of the TML prodrug nanoparticles was a hundredfold higher than that of Azopt. This suggests that nano eye-drops based on TML prodrug should be far superior to Azopt in clinical use.

### Hydrolysis rate of the brinzolamide prodrug nanoparticles

**Figure 3:**
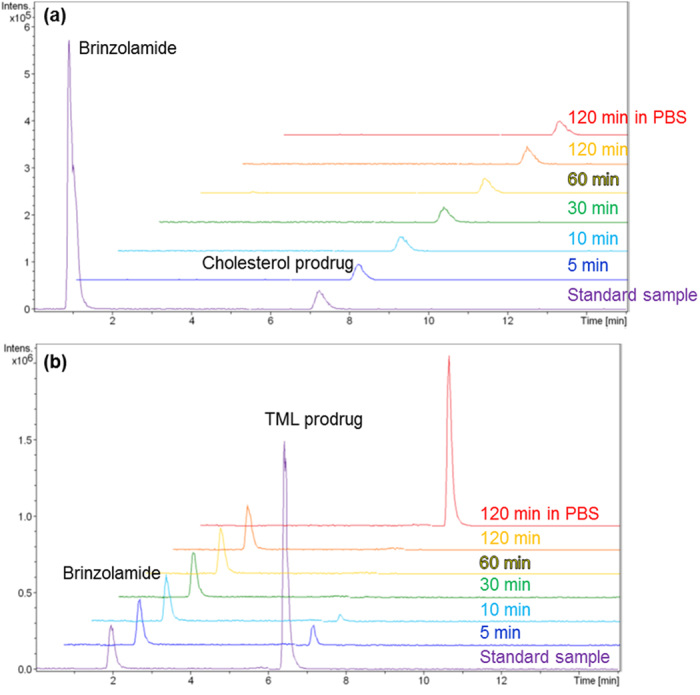
Hydrolysis rates for cholesterol prodrug nanoparticles (**a**) and TML prodrug nanoparticles (**b**). Cholesterol prodrug nanoparticles were not hydrolysed by enzymes present in the aqueous humour, whereas TML prodrug nanoparticles immediately released brinzolamide.

In order to exert their ocular hypotensive effects, nanoparticles consist of brinzolamide prodrugs must be hydrolysed to release brinzolamide. Elucidation of the hydrolytic behaviours of these nanoparticles is critical for preparation of the effective nano eye-drops. Therefore, we evaluated the hydrolysis rate of brinzolamide prodrug nanoparticles in the harvested aqueous humour of rats. Aqueous humour is the liquid component between the cornea and lens and contains the enzyme needed to hydrolyse the prodrug^47^,^48^. Unfortunately, nanoparticles of the cholesterol prodrug were not hydrolysed in the aqueous humour (Fig. 3a). This may be because cholesterol is a bulky substituent. Since the hydrolysis site was located between brinzolamide and cholesterol, we assumed that the enzyme may not have had sufficient access to the hydrolysable carbamate bonds of the cholesterol prodrug. However, TML prodrug nanoparticles were immediately hydrolysed by the enzyme present in the aqueous humour and could release some of the bound brinzolamide, as shown in Fig. 3b. Until 30 min, all TML prodrug nanoparticles were stoichiometrically converted to brinzolamide. No degradation of TML prodrug in PBS supported the significant role of the enzyme in proposed DDSs. We expected that the TML-substituted prodrug was easily hydrolysed by the enzyme because the first site of hydrolysis was located at the end of the molecule. The second intramolecular hydrolysis reaction could then proceed rapidly. In other words, control of the hydrolysis rate could be achieved by changing the structure and design of the prodrug.

### Ocular hypotensive effects of brinzolamide prodrug nano eye-drops

**Figure 4:**
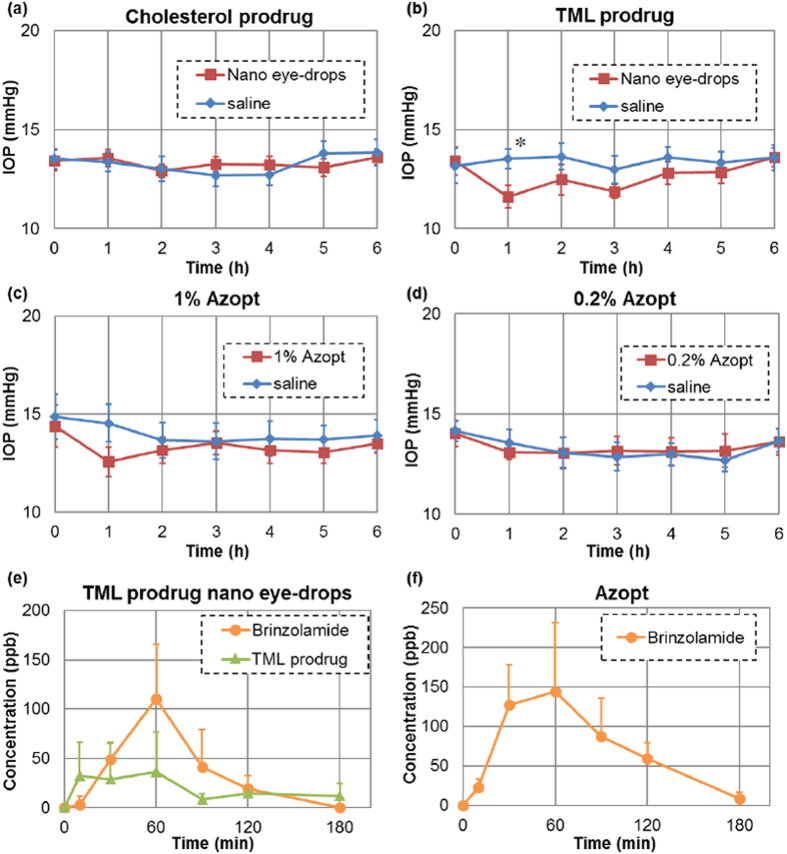
Ocular hypotensive effect of cholesterol prodrug nano eye-drops (**a**), TML prodrug nano eye-drops (**b**), 1% Azopt (**c**), and 0.2% Azopt (**d**). The data show the means ± SEs (n = 6). The ocular hypotensive effect of nano eye-drops was closely related to the hydrolysis rate. TML prodrug nano eye-drops showed effects similar to those of Azopt. This result agreed with the ocular penetration rate of TML prodrug nano eye-drops (**e**) and Azopt (**f**). Data show the means + SDs (n = 6). *P < 0.05, significantly different from saline.

The ocular hypotensive effects of these nano eye-drops were evaluated by administration of nano eye-drops of brinzolamide prodrug in anesthetised Sprague-Dawley (SD) rats with normal IOP. An aqueous dispersion of the brinzolamide prodrug was mixed with 9% NaCl aqueous solution (9:1) to maintain the concentration of NaCl in the eye-drops. Figure 4a shows time-dependent changes in IOP when the cholesterol prodrug nano eye-drops and saline as a control were administered to the right and left eyes of SD rats, respectively. Cholesterol prodrug nano eye-drops did not cause an ocular hypotensive effect because the hydrolysis reaction did not occur. Figure 4b shows time-dependent changes in IOP when TML prodrug nano eye-drops and saline were added to right and left eyes of SD rats, respectively. In this experiment, we observed an obvious ocular hypotensive effect in eyes treated with the nano eye-drops containing the TML prodrug. In addition, the potency of TML prodrug nano eye-drops was almost equivalent to that of Azopt (Fig. 4c). Indeed, 5.67 mM TML prodrug nano eye-drops were as effective as 26.1 mM Azopt (Fig. 4b and c), demonstrating that TML prodrug nano eye-drops were sufficiently effective at one-fifth the concentration of Azopt. Moreover, when the concentration of Azopt was reduced to one-fifth that of the original solution (i.e., 0.2%), no ocular hypotensive effects were observed (Fig. 4d). These data indicated that TML prodrug nano eye-drops were around five times more effective than Azopt.

### Ocular penetration rate of brinzolamide prodrug nano eye-drops

Brinzolamide acts as a carbonic anhydrase inhibitor at the ciliary body, and inhibition of aqueous humour production induces a decrease in the IOP^39^. The concentration of brinzolamide in the aqueous humour is closely related to the ocular hypotensive effects of eye-drops because the ciliary body is in contact with aqueous humour, and brinzolamide can reach the site through the aqueous humour^24^. Therefore, we evaluated the concentration of TML prodrug and brinzolamide in aqueous humour of rats. After administration of TML prodrug nano eye-drops, the amount of brinzolamide in the aqueous humour increased at 60 min, as shown in Fig. 4e. This result was consistent with the ocular hypotensive effects of TML prodrug nano eye-drops (Fig. 4b). The IOP of rats treated with TML prodrug nano eye-drops was decreased after 1 h and then returned to baseline. Nonhydrolysed TML prodrug was also observed in the aqueous humour. In the case of Azopt, the concentration of brinzolamide in the aqueous humour reached a maximum at 60 min, similar to TML prodrug nano eye-drops (Fig. 4f), and this was approximately equal amount to TML prodrug nano eye-drops treatment.

Our IOP and ocular penetration rate results could be explained by effects of nanoparticles. As mentioned in the Introduction part, nanoparticles are able to easily permeate the cornea compared with molecules and microparticles because of their size and hydrophobic properties^26^. Brinzolamide dispersed in Azopt cannot easily penetrate into the eye through the cornea because of its micro-sized fibre-like structure. On the other hand, the TML prodrug present in the nano eye-drops formed nano-sized particles. Therefore, TML prodrug nano eye-drops could easily penetrate into the cornea and effectively decrease the IOP of rats at lower brinzolamide concentrations than that found in Azopt.

### Assessment of the toxicity of the TML prodrug nano eye-drops using histological and biochemical evaluation after topical ocular treatment

**Figure 5:**
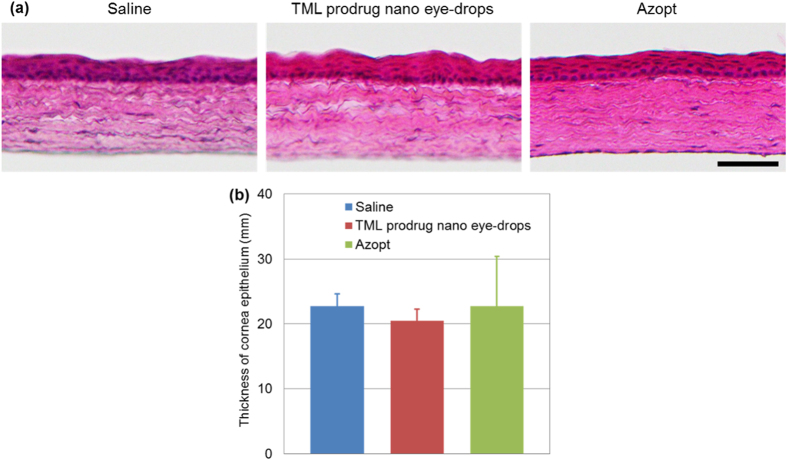
Histological analysis of rat cornea tissues after treatment with TML prodrug nano eye-drops. (**a**) H&E staining of corneal cryosections after 1 week of topical ocular treatment with saline (vehicle), TML prodrug nano eye-drops, or Azopt. Scale bar, 50 μm. (**b**) Histogram showing the average thickness of rat corneas after treatment with saline, TML prodrug nano eye-drops, or Azopt. The histogram data are showed means ± SDs (n = 4).

To test whether the nano eye-drops was toxic, we examined histological sections of the rat cornea after treatment with the TML prodrug nano eye-drops. Although the chemical structure of the TML prodrug was modified from the original brinzolamide compound that is already used for clinical applications, it is necessary to evaluate the toxicology of all new chemical compounds or formulations. In histological analysis by hematoxylin and eosin (H&E) staining, there were no toxicological features observed in the rat cornea including the epithelium and the stroma at 1 week after ocular treatment with TML prodrug nano eye-drops as compared with saline treatment or Azopt treatment as controls (Fig. 5a). The thickness of the rat corneal epithelium at 1 week after topical ocular application of TML prodrug nano eye-drops was not significantly different from that observed after vehicle treatment or Azopt treatment (saline: 22.7 ± 1.9 μm; TML prodrug nano eye-drops: 20.5 ± 1.9 μm; Azopt: 22.7 ± 7.7 μm; Fig. 5b). Thus, histological analysis suggested that the TML prodrug nano eye-drops did not cause damage to rat corneal tissue.

**Figure 6:**
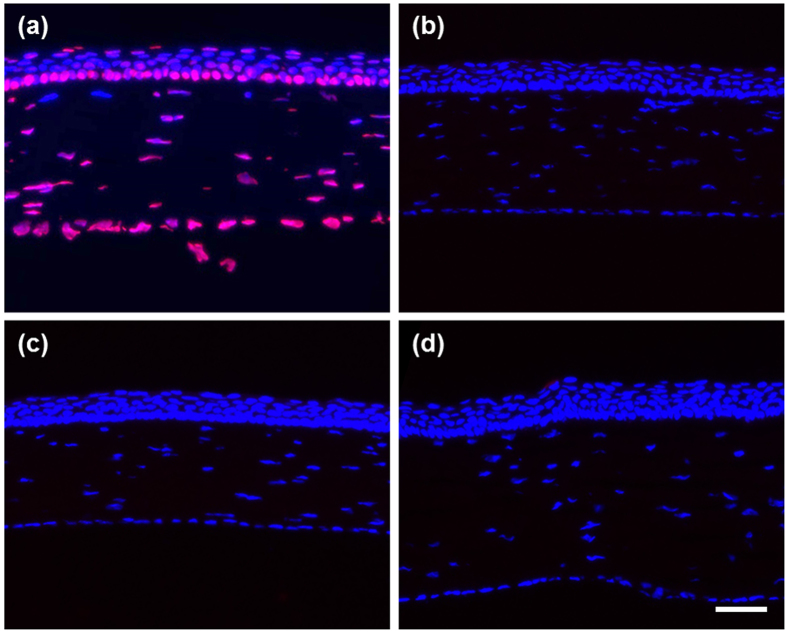
Detection of apoptotic cell death in rat corneas after treatment with nano eye-drops. (**a**) Fragmented DNA induced by DNaseI treatment was detected by TUNEL (red) as a positive control. Nuclei were counterstained with DAPI (blue). Rat cornea sections were from saline treatment (**b**), TML prodrug nano eye-drops treatment (**c**), or Azopt treatment (**d**). Scale bar, 50 μm.

TUNEL was then performed to determine whether apoptotic cell death was induced after treatment with the nano eye-drops^49^,^50^. TUNEL-positive signals were detected in the sections of rat cornea treated with DNaseI, indicating that the experimental procedure and reagents were working properly (Fig. 6a). Meanwhile, in the sections from rats treated with TML prodrug nano eye-drops for 1 week, TUNEL-positive cells in the cornea epithelium were rarely observed (0.0139 ± 0.028 cells/eye, n = 4), similar to sections from rats treated with 0.9% NaCl (0.0139 ± 0.028 cells/eye, n = 4) or Azopt (0.0278 ± 0.056 cells/eye, n = 4) as controls (Fig. 6b–d). These results indicated that topical ocular treatment with the TML prodrug nano eye-drops did not induce apoptosis in the rat corneal epithelium *in vivo*, similar to treatment with Azopt.

## Discussion

Nanoparticles of brinzolamide were successfully obtained by modification of the hydrophobic substituent. Cholesterol prodrug nanoparticles were not hydrolysed by enzyme present in the aqueous humour owing to their bulky hydrophobic substituents. However, TML prodrug nanoparticles were immediately hydrolysed in the aqueous humour because of a novel trimethyl locked structure. The hydrolytic behaviour of brinzolamide prodrug nanoparticles was closely related to the ocular hypotensive effects of the resulting nano eye-drops. TML prodrug nanoparticles in eye-drops would penetrate into the eye and effectively decrease the IOP. Notably, TML prodrug nano eye-drops were as effective as Azopt at one-fifth the molar concentration and did not cause any toxic effects in the cornea. In addition, the high light transmittance of the aqueous dispersion of the TML prodrug nanoparticles will lead to improvement of current patient discomfort after administration. Thus, preparation of nano eye-drops may represent the next generation of ophthalmological therapeutics.

## Methods

### General procedures

All reactions were carried out in flame-dried glassware under a nitrogen atmosphere with dry solvents. Reactions were monitored by analytical thin layer chromatography (TLC) carried out on 0.25 mm silica gel plates. Visualisation of the developed plates was performed using UV absorbance and aqueous cerium ammonium molybdate. Flash chromatography was performed on silica gel with the indicated solvent systems. IR spectra were recorded on a Thermo Scientific NICOLET 380 system. NMR spectra were recorded on JEOL JNM-AL400 spectrometer or JEOL ECA-700 spectrometer and calibrated using residual undeuterated solvent as an internal reference (CDCl_3_ at *δ* 7.26 ppm for ^1^H and *δ* 77.2 ppm for ^13^C NMR). Mass spectra were obtained with a SHIMADZU GCMS-QP2010 using electron ionization (EI). HRMS was performed using a micrOTOF-Q II-S1 with electrospray ionisation (ESI). Zeta potential and the size distribution of nanoparticles in aqueous dispersion were measured using a Malvern Zetasizer nanoZS. Scanning electron microscopy (SEM) images were obtained using a JEOL JSM-6700F instrument. Transmittance of aqueous dispersion was measured using a UV-visible spectrophotometer (JASCO, V-570). Liquid chromatography-tandem mass spectrometry (LC-MS/MS) was performed using high-performance liquid chromatography (HPLC; Agilent 1260 Infinity) connected to a mass spectrometer (Bruker HCT ultra-IMR).

### Materials

4-Dimethylaminopyridine (DMAP), trimethylamine (NEt_3_), deuterochloroform (CDCl_3_), and sodium chloride (NaCl) were purchased from Wako Pure Chemical Industries, Ltd. Isobutyl chloroformate, pyridinium chlorochromate (PCC), and 1-ethyl-3-(3-dimethylaminopropyl)carbodiimide hydrochloride (EDC) were purchased from Tokyo Chemical Industry Co., Ltd. Tetrahydrofuran (THF), hydrochloric acid (HCl), ammonium chloride (NH_4_Cl), potassium carbonate (K_2_CO_3_) and acetic acid (AcOH) were purchased from Junsei Chemical Co., Ltd. *t*-Butyldimethylsilyl chloride, iodomethane (CH_3_I), potassium permanganate (KMnO_4_), dichloromethane (CH_2_Cl_2_), hexane, ethyl acetate (AcOEt), methanol (MeOH), acetonitrile (CH_3_CN), sodium bicarbonate (NaHCO_3_), anhydrous magnesium sulphate (MgSO_4_), and silica gel were purchased from Kanto Chemical Co., Ltd. Acetone was purchased from Daishin Chemical Co., Ltd. Brinzolamide was purchased from Yitai Pharmatec Co., Ltd. Isoflurane was purchased from Pfizer Co., Ltd. as Escain. Sevoflurane was purchased from Maruishi Pharmaceutical Co., Ltd. as Sevofrane. Ketamine hydrochloride was purchased from Daiichi-Sankyo Co., Ltd. as KETALAR. Xylazine hydrochloride was purchased from Bayer HealthCare Co., Ltd. as Selactar. Azopt was purchased from Alcon Co., Ltd. These reagents were used without further purification.

### Animals

Specific-pathogen free male Sprague-Dawley (SD) rats were obtained from Japan SLC Inc. The Ethical Committee of the Graduate School of Medicine, Tohoku University, approved the protocol as #2014MdA-084. The methods were carried out in accordance with the approved guidelines.

### Fabrication of brinzolamide micro-sized fibres and TML prodrug nanoparticles

Brinzolamide micro-sized fibres and brinzolamide prodrug nanoparticles were prepared using the reprecipitation method. At ambient temperature, 25 mg/mL brinzolamide and 50 mg/mL polysorbate 80 in acetone (200 μL) were injected into stirring distilled water (800 μL).

At ambient temperature, 25 mg/mL TML prodrug and 50 mg/mL polysorbate 80 in acetone (200 μL) were injected into stirring distilled water (800 μL) in an ice bath, and the resulting aqueous dispersion was stirred for 10 min in an ice bath. After reprecipitation, an aqueous dispersion of TML prodrug nanoparticles was dialysed twice using dialysis membrane (Thermo Fisher Scientific Inc., MWCO: 12,000–14,000) to remove acetone.

### Liquid chromatography tandem mass spectrometry (LC-MS/MS)

The HPLC conditions for the TML prodrug were as follows: column, reverse-phase C_8_ column (Imtakt Unison UK-C_8_, *ø* 2 × 100 mm); column temperature, 35 °C; mobile phase, gradient generated from MeOH with 0.1% formic acid/water with 0.1% formic acid (v/v) = 30/70 to 80/20 at 1 min; flow rate, 0.3 mL/min; injection volume, 1 μL; retention time for brinzolamide, 1.9 min; and retention time for TML prodrug, 6.4 min. Precursor ions for brinzolamide (*m/z* 384.00) and TML prodrug (*m/z* 794.20) were used^51^.

The HPLC conditions for Azopt were as follows: column, reverse-phase ODS column (Imtakt Cadenza CD-C_18_, *ϕ* 2 × 100 mm); column temperature, 35 °C; mobile phase, MeOH with 0.1% formic acid/water with 0.1% formic acid (v/v) = 30/70; flow rate, 0.3 mL/min; injection volume, 1 μL; retention time for brinzolamide, 2.1 min. Precursor ions for brinzolamide (*m/z* 384.00) was used.

### Hydrolysis of brinzolamide prodrug nanoparticles

Aqueous humour was collected from SD rats (12–15 weeks of age) under anaesthesia using sevoflurane and a mixture of ketamine hydrochloride (160 mg/kg body weight) and xylazine hydrochloride (16 mg/kg body weight).

The hydrolysis rate of TML prodrug nanoparticles in aqueous humour was evaluated as follows. First, a 0.01% aqueous dispersion of TML prodrug nanoparticles (15 μL) was added to aqueous humour (135 μL). The mixture was heated at 37 °C in a water bath. After 5, 10, 30, 60, or 120 min, the reaction mixture (10 μL) was added to MeOH (90 μL) and centrifuged at 10000 rpm for 5 min. The supernatant was diluted by a factor of 10 using a 9:1 mixture of MeOH and distilled water, and the hydrolysis rate of the samples was evaluated using LC-MS/MS.

The hydrolysis rate of TML prodrug nanoparticles in PBS was evaluated as follows. First, a 0.01% aqueous dispersion of TML prodrug nanoparticles (15 μL) was added to PBS (135 μL). The mixture was heated at 37 °C in a water bath. After 120 min, the reaction mixture (10 μL) was added to MeOH (90 μL) and centrifuged at 10000 rpm for 5 min. The supernatant was diluted by a factor of 10 using a 9:1 mixture of MeOH and distilled water, and the hydrolysis rate of the samples was evaluated using LC-MS/MS.

### Evaluation of intraocular pressure (IOP)

The aqueous dispersion of TML prodrug nanoparticles (90 μL) was added to a 9% aqueous solution of NaCl (10 μL) in order to obtain the nano eye-drops. SD rats (10–12 weeks) were anaesthetised with 5% isoflurane and maintained by 2% isoflurane during measurement of IOP. IOP was measured by icare six times. Nano eye-drops, 1% or 0.2% Azopt (10 μL) were then administrated to the right eye, and saline (10 μL) was administered to left eye as a control. The IOPs of both eyes were measured under anaesthesia every hour for up to 6 h.

### Statistical Analysis

Statistical comparisons were performed using an independent samples Student’s t test. The results were considered statistically significant at *P* < 0.05.

### Ocular penetration rate

SD rats (11–23 weeks of age) were anaesthetised with 5% isoflurane, and TML prodrug nano eye-drops or Azopt (10 μL) were administered. After 10, 30, 60, 90, 120, or 180 min, rats were anaesthetised using sevoflurane and a mixture of ketamine hydrochloride (160 mg/kg body weight) and xylazine hydrochloride (16 mg/kg body weight). Eye of rats were washed with saline (20 μL × 2), and aqueous humour (15 μL) was harvested. The resulting aqueous humour was added to MeOH (135 μL) and centrifuged at 10000 rpm for 5 min. The ocular penetration rate of the samples was evaluated using LC-MS/MS.

### Topical ocular treatment for toxicological study

The aqueous dispersion of TML prodrug nanoparticles (90 μL) was added to a 9% aqueous solution of NaCl (10 μL) in order to obtain the nano eye-drops. Sodium chloride (0.9%) served as the vehicle control, and Azopt was the control for the original chemical compound. TML prodrug nano eye-drops, saline, and Azopt were applied topically to the right eyes of male SD rats (n = 4 per group) one time daily for 7 days.

### Hematoxylin and eosin (H&E) staining

H&E staining of rat cornea cryosections was performed as described previously with minor modifications. Hematoxylin staining was performed with hematoxylin solution (Type M, Muto Pure Chemicals, Tokyo, Japan) for 1 min, and sections were then stained for 30 s with 0.3% eosin alcohol solution (Muto Pure Chemicals). Images of stained rat cornea sections were captured with an Olympus BX53 microscope using a 10 × objective lens and software (Standard Cellsens, Olympus, Tokyo, Japan).

### Cryosections and terminal deoxynucleotidyl transferase-mediated dUTP nick end labelling (TUNEL)

Rat corneas were embedded in OCT compound (Sakura Finetechnical, Tokyo, Japan). Cryosections (12 μm) were cut on a cryostat CM3050 (Leica Microsystems GmbH, Wetzlar, Germany) and were used for TUNEL to detect apoptotic cells. TUNEL was performed using an ApopTag Red *In Situ* Apoptosis Detection Kit (Chemicon International, Inc., Temecula, CA, USA) according to the manufacturer’s instruction. Sections were mounted, and nuclei were stained with 4′-6′-diamidino-2-phenylindole (DAPI) using Vectashield (H-1200; Vector, Burlingame, CA, USA). The cryosections were incubated with 4000 U/mL DNase1 (Sigma-Aldrich, St. Louis, MO, USA) in 50 mM Tris-HCl (pH 7.5) containing 1 mg/mL BSA (Sigma-Aldrich) for 10 min at room temperature as the TUNEL-positive control. Fluorescence images of whole rat corneas were acquired, and TUNEL-positive cells were counted using a BZ-X710 fluorescence microscope (Keyence, Osaka, Japan). Representative fluorescence images were captured with an Axiovert 200 (Carl Zeiss AG, Feldbach, Switzerland).

## Additional Information

**How to cite this article**: Ikuta, Y. *et al*. Creation of nano eye-drops and effective drug delivery to the interior of the eye. *Sci. Rep.* **7**, 44229; doi: 10.1038/srep44229 (2017).

**Publisher's note:** Springer Nature remains neutral with regard to jurisdictional claims in published maps and institutional affiliations.

## Supplementary Material

Supplementary Information
