# Supplementary material for: Creation of nano eye-drops and effective drug delivery to the interior of the eye
Source: Sci Rep. 2017 Mar 14;7:44229. doi: 10.1038/srep44229 (PMC5349510; doi:10.1038/srep44229)
Supplement: Supplementary Information [file srep44229-s1.pdf]

Supplementary Information

Creation of nano eye-drops and effective drug delivery to the interior of the eye

*Yoshikazu Ikuta, Shigenobu Aoyagi, Yuji Tanaka, Kota Sato, Satoshi Inada, Yoshitaka Koseki, Tsunenobu Onodera, Hidetoshi Oikawa, Hitoshi Kasai**

Synthesis of TML prodrug

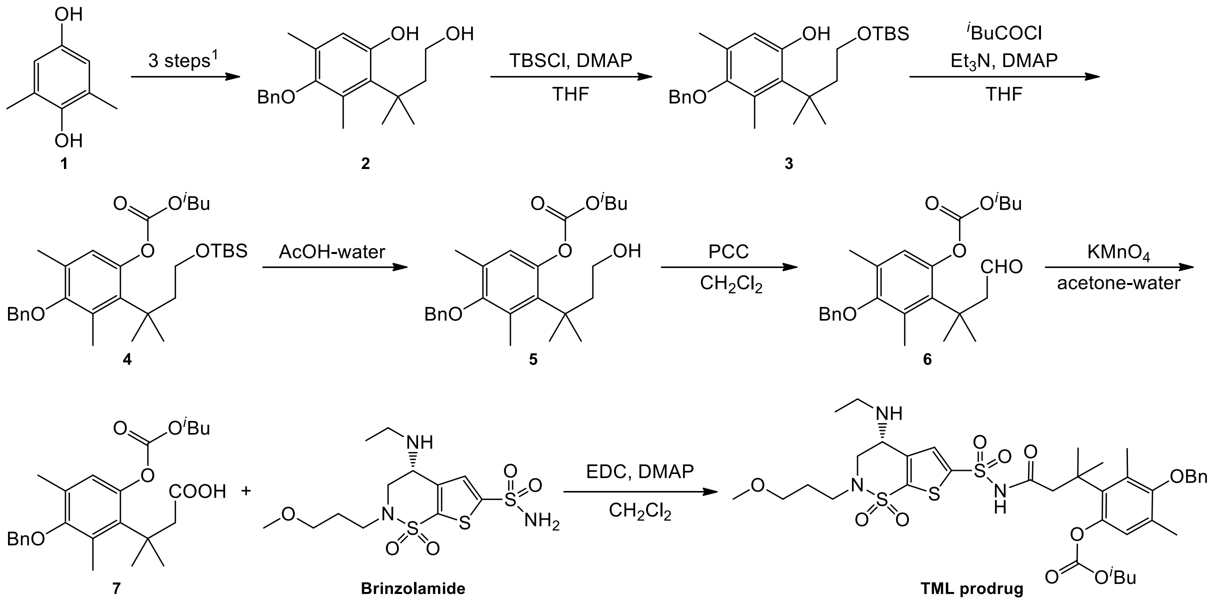

Supplementary Figure 1. Synthesis of the TML prodrug.

Compound **2** was synthesised as previously described[1].

1-O-*tert*-Butyldimethylsilyl-3-(2′-hydroxy-4′,6′-dimethyl-5′-benzyloxyphenyl)-3,3-dimethylpropanol (**3**)

t-Butyldimethylsilyl chloride (9.75 g, 64.7 mmol) was added to a solution of **2** (16.9 g, 53.7 mmol) and DMAP (9.84 g, 80.5 mmol) in anhydrous THF (200 mL) at 0–5°C. The reaction was stirred for 20 h at room temperature. The mixture was then extracted with AcOEt (200 mL), washed with water (200 mL) and brine (100 mL × 3), and dried over anhydrous $MgSO_4$. The solution was concentrated under reduced pressure to afford 23.0 g (98%) of **3** as a colourless oil. IR (oil): ν_{max} 3502, 2954, 2926, 2855, 1713, 1471, 1454, 1403, 1365, 1252, 1225, 1191, 1097, 1036, 1026, 859, 836, 776, 734, 697 cm^{-1}; ^{1}H NMR (400 MHz, $CDCl_3$): δ 0.02 (s, 6H), 0.87 (s, 9H), 1.58 (s, 6H), 2.09 (t, J = 6.6 Hz, 2H), 2.22 (s, 3H), 2.42 (s, 3H), 3.63 (t, J = 6.6 Hz, 2H), 4.70 (s, 2H), 5.84 (s, 1H), 6.46 (s, 1H), 7.33 (t, J = 7.2 Hz, 1H), 7.39 (t, J = 7.2 Hz, 2H), 7.47 (d, J = 7.2 Hz, 2H); ^{13}C NMR (101 MHz, $CDCl_3$): δ −5.13, 16.3,

16.5, 18.4, 26.1, 32.5, 40.0, 44.9, 62.0, 74.1, 117.8, 127.55, 127.64, 128.3, 128.8, 131.0, 131.1, 137.7, 149.8, 151.6; EI-MS (70 eV) *m/z* calc'd. for $C_{26}H_{40}O_3Si$ $[M]^+$ 428, found 428 (0.4) $[M]^+$, 205 (100) $[M{-}TBS{-}OBn]^+$.

1-O-*tert*-Butyldimethylsilyl-3-(2′-*i*-butoxycarbonyloxy-4′,6′-dimethyl-5′-benzyloxyphenyl)-3,3-dimethylpropanol (**4**)

Isobutyl chloroformate (9.86 g, 80.5 mmol) was added to a solution of **3** (23.0 g, 53.7 mmol), NEt_3 (10.9 g, 107 mmol), and DMAP (1.31 g, 10.7 mmol) in THF (200 mL) at 0–5°C, and the reaction mixture was stirred for 3 h at room temperature. The reaction mixture was then diluted with AcOEt (200 mL), washed with water (200 mL) and brine (100 mL × 3), and dried over anhydrous $MgSO_4$. The solution was concentrated under reduced pressure to afford 28.4 g of **4** as a colourless solid quantitatively. IR (oil): ν_{max} 2958, 2928, 2855, 1758, 1471, 1369, 1243, 1208, 1090, 996, 972, 835, 776, 733, 696 cm^{-1}; ^1H NMR (400 MHz, $CDCl_3$): δ 0.02 (s, 6H), 0.85 (s, 9H), 1.00 (d, J = 6.6 Hz, 6H), 1.51 (s, 6H), 2.00–2.09 (m, 1H), 2.07 (t, 2H, J = 7.6 Hz), 2.26 (s, 3H), 2.49 (s, 3H), 3.52 (t, J = 7.6 Hz, 2H), 4.02 (d, 2H, J = 6.6 Hz), 4.74 (s, 2H), 6.70 (s, 1H), 7.34 (t, J = 7.2 Hz, 1H), 7.40 (t, J = 7.2 Hz, 2H), 7.47 (d, J = 7.2 Hz, 2H); ^{13}C NMR (101 MHz, $CDCl_3$): δ −5.12, 16.3, 16.6, 18.3, 19.0, 26.0, 27.9, 31.8, 40.0, 45.8, 60.8, 73.7, 74.5, 123.7, 127.5, 127.8, 128.3, 129.5, 131.9, 136.0, 137.3, 145.8, 154.21, 154.27; EI-MS (70 eV) *m/z* calc'd. for $C_{31}H_{48}O_5Si$ $[M]^+$ 528, found 471 (14) $[M{-}Bu]^+$, 205 (29) $[M{-}^iBuOCO{-}TBS{-}OBn]^+$, 91 (100) $[Bn]^+$.

3-(2′-*i*-Butoxycarbonyloxy-4′,6′-dimethyl-5′-benzyloxyphenyl)-3,3-dimethylpropanol (**5**)

AcOH (160 mL) and water (160 mL) were added to a solution of **4** (28.4 g, 53.7 mmol) in THF (250 mL), and the solution was then stirred for 4 h at 40°C. The reaction mixture was diluted with AcOEt (200 mL); washed with water (200 mL), saturated aqueous solution of $NaHCO_3$ (100 mL × 3), and brine (200 mL); and dried over anhydrous $MgSO_4$. The solution was concentrated under reduced pressure to afford 22.3 g of **5** as a colourless solid quantitatively. IR (oil): ν_{max} 3468, 2959, 2928, 2874, 1756, 1470, 1454, 1402, 1370, 1244, 1209, 996, 971, 782, 735, 697 cm^{-1}; ^1H NMR (400 MHz, $CDCl_3$): δ 1.00 (d, J = 6.6 Hz, 6H), 1.52 (s, 6H), 2.00–2.07 (m, 1H), 2.10 (t, J = 7.2 Hz, 2H), 2.26 (s, 3H), 2.48 (s, 3H), 3.57 (t, J = 7.6 Hz, 2H), 4.03 (d, J = 6.6 Hz, 2H), 4.75 (s, 2H), 6.71 (s, 1H), 7.35 (t, J = 7.2 Hz, 1H), 7.40 (t, J = 7.2 Hz, 2H), 7.46 (d, J = 7.2 Hz, 2H); ^{13}C NMR (101 MHz, $CDCl_3$): δ 16.3, 16.6, 19.0, 27.9, 31.9, 39.6, 45.7, 60.5, 73.7, 74.7, 123.9, 127.5, 127.8, 128.3, 129.8, 132.1, 135.6, 137.2, 145.7, 154.38, 154.47; EI-MS (70 eV) *m/z* calc'd. for $C_{25}H_{34}O_5$ $[M]^+$ 414, found 414 (0.01) $[M]^+$, 205 (100) $[M-^iBuOCO-OBn]^+$.

3-(2′-*i*-Butoxycarbonyloxy-4′,6′-dimethyl-5′-benzyloxyphenyl)-3,3-dimethylpropanal (**6**)

PCC (17.4 g, 80.7 mmol) was added to a solution of **5** (22.3 g, 53.7 mmol) in CH_2Cl_2 (150 mL) at 0–5°C, and the mixture was stirred for 2 h at room temperature. Silica gel was added to the reaction mixture, and the solvent was removed by evaporation. The residue was purified by column chromatography on silica gel with eluent hexane/AcOEt (4:1) to afford 17.8 g (92 %) of **6** as a pale yellow oil. IR (oil): ν_{max} 2961, 2930, 2874, 1758, 1713, 1470, 1453, 1370, 1241, 1210, 1001, 970, 781, 735, 697 cm^{-1}; ^1H NMR (400 MHz, $CDCl_3$): δ 1.00 (d, J = 6.4 Hz, 6H), 1.51 (s, 6H), 2.01–2.08 (m, 1H), 2.26 (s, 3H), 2.47 (s, 3H), 2.87 (s, 2H), 4.04 (d, J = 6.4 Hz, 2H), 4.74 (s, 2H), 6.76 (s, 1H), 7.34 (t, J = 7.2 Hz, 1H), 7.40 (t, J = 7.2 Hz, 2H), 7.46 (d, J = 7.2 Hz, 2H), 9.55 (t, J = 2.6 Hz, 1H); ^{13}C NMR (101 MHz, $CDCl_3$): δ 16.3, 16.7, 19.0, 27.9, 31.5, 38.6, 56.7, 73.8, 74.7, 123.9, 127.5, 127.8, 128.3, 130.4, 131.5,

134.4, 137.1, 145.3, 153.9, 154.5, 202.2; EI-MS (70 eV) *m/z* calc'd. for $C_{26}H_{32}O_5$ $[M]^+$ 412, found 412 (0.02) $[M]^+$, 91 (100) $[Bn]^+$.

3-(2′-*i*-Butoxycarbonyloxy-4′,6′-dimethyl-5′-benzyloxyphenyl)-3,3-dimethylpropionic acid (**7**)

$KMnO_4$ (6.82 g, 43.1 mmol) in 250 mL water was added to a solution of **6** (17.8 g, 43.1 mmol) in acetone (250 mL) at 0–5°C, and the mixture was stirred for 17 h at room temperature. The reaction mixture was filtered to remove inorganic solids, and the solvent was removed by evaporation. The residue was dissolved in AcOEt (200 mL), washed with 3N HCl (200 mL) and brine (200 mL × 3), and dried over anhydrous $MgSO_4$. The solution was concentrated under reduced pressure and purified by column chromatography on silica gel with eluent hexane/AcOEt (2:3) to afford 12.0 g (72%) of **7** as a colourless oil. IR (oil): ν_{max} 3031, 2961, 2928, 2874, 1758, 1707, 1470, 1454, 1370, 1266, 1242, 1211, 996, 972, 780, 734, 698 cm^{-1}; ^1H NMR (400 MHz, $CDCl_3$): δ 0.99 (d, J = 6.8 Hz, 6H), 1.60 (s, 6H), 2.04 (septet, J = 6.8 Hz, 1H), 2.23 (s, 3H), 2.48 (s, 3H), 2.89 (s, 2H), 4.02 (d, J = 6.8 Hz, 2H), 4.73 (s, 2H), 6.72 (s, 1H), 7.33 (t, J = 7.2 Hz, 1H), 7.38 (t, J = 7.2 Hz, 2H), 7.45 (d, J = 7.2 Hz, 2H); ^{13}C NMR (101 MHz, $CDCl_3$): δ 16.3, 16.5, 19.0, 27.9, 31.3, 39.2, 47.4, 73.8, 74.6, 123.6, 127.5, 127.7, 128.3, 129.8, 131.6, 135.2, 137.2, 145.5, 154.0, 154.2, 177.2; HRMS (ESI) *m/z* calc'd. for $C_{25}H_{32}O_6Na$ $[M+Na]^+$ 451.2081, found 451.2064.

Preparation of TML prodrug

EDC (2.00 g, 10.4 mmol) and DMAP (63.8 mg, 0.52 mmol) were added to a solution of brinzolamide (2.00 g, 5.22 mmol) and **7** (3.36 g, 7.83 mmol) in CH_2Cl_2 (45 mL) at 0–5°C, and the reaction mixture was stirred for 18 h at room temperature. The resultant mixture was diluted with $CHCl_3$ (25 mL). The solution was washed with saturated aqueous solution of $NaHCO_3$, saturated aqueous solution of NH_4Cl, and water and dried over anhydrous $MgSO_4$.

The solution was concentrated under reduced pressure and purified with chromatography on silica gel with eluent AcOEt/MeOH (50:1) followed by removal of solvent *in vacuo* to afford 2.55 g (66%) of TML prodrug as a pale yellow solid. IR (KBr): ν_{max} 3422, 2966, 2924, 2875, 1759, 1727, 1590, 1471, 1360, 1247, 1165, 1135, 1012, 950, 823, 785, 671, 651, 619, 588, 566 cm^{-1}; ^{1}H NMR (400 MHz, $CDCl_3$): δ 1.03 (d, J = 6.8 Hz, 6H), 1.13 (t, J = 6.8 Hz, 3H), 1.62 (s, 3H), 1.63 (s, 3H), 1.93 (quintet, J = 6.8 Hz, 2H), 2.10 (septet, J = 6.8 Hz, 1H), 2.23 (s, 3H), 2.28 (s, 3H), 2.45 (s, 2H), 2.73 (dq, J = 1.6, 6.8 Hz, 2H), 3.27–3.32 (m, 1H), 3.34 (s, 3H), 3.46–3.52 (m, 2H), 3.56–3.63 (m, 1H), 3.86 (t, J = 5.6 Hz, 2H), 3.92–3.96 (m, 1H), 4.14 (d, J = 6.8 Hz, 2H), 6.69 (s, 1H), 6.80 (s, 1H), 7.75 (s, 1H); ^{13}C NMR (101 MHz, $CDCl_3$): δ 15.8, 18.9, 20.3, 25.5, 27.9, 29.7, 32.3, 40.7, 41.4, 46.6, 50.2, 50.3 , 51.5, 58.8, 69.2, 75.8, 122.8, 131.3, 133.56, 133.64, 137.9, 138.2, 140.3, 142.3, 143.5, 150.2, 156.0, 169.1; HRMS (ESI) *m/z* calc'd. for $C_{37}H_{52}N_3O_{10}S_3$ $[M+H]^+$ 794.2809, found 794.2816.

Dimethylation of TML prodrug

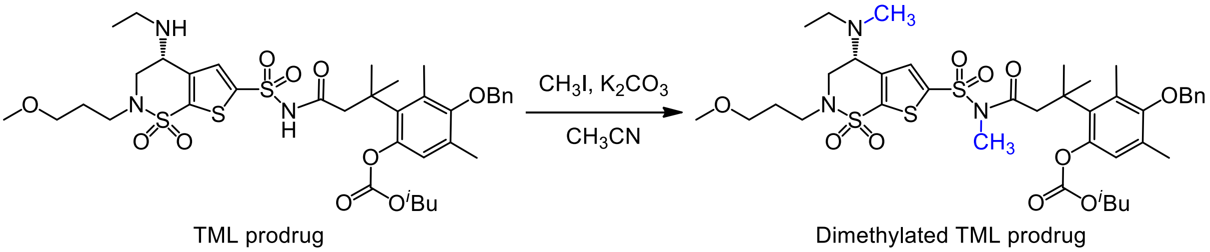

Supplementary Figure 2. Synthesis of dimethylated TML prodrug.

A suspension of TML prodrug (100 mg, 0.126 mmol), CH_3I (107 mg, 0.756 mmol), and K_2CO_3 (87.0 mg, 0.630 mmol) in CH_3CN (10 mL) was refluxed for 4 h. The hot suspension was filtered to remove inorganic solid, and the solvent was removed by evaporation. The residue was dissolved in CH_2Cl_2 (10 mL) and washed with water (10 mL). The aqueous layer was extracted with CH_2Cl_2 (10 ml × 3), and the combined organic layer was washed with a saturated aqueous solution of $NaHSO_3$ and dried over anhydrous $MgSO_4$. The solution was concentrated under reduced pressure and purified with chromatography on silica gel with AcOEt as the eluent followed by removal of solvent *in vacuo* to afford 57.1 mg (55%) of dimethylated TML prodrug as a colourless oil. IR (oil): ν_{max} 2966, 2932, 2876, 1758, 1720, 1470, 1370, 1241, 1214, 1170, 1076, 912, 863, 733, 653, 617, 588 cm^{-1}; ^{1}H NMR (700 MHz, $CDCl_3$): δ 1.00 (d, J = 7.0 Hz, 6H), 1.05 (t, J = 7.0 Hz, 3H), 1.56 (s, 3H), 1.57 (s, 3H), 1.91 (quintet, J = 7.0 Hz, 2H), 2.05 (septet, J = 7.0 Hz, 1H), 2.22 (s, 3H), 2.24 (s, 3H), 2.48 (s, 3H), 2.51 (dq, J = 2.1, 7.0 Hz, 2H), 3.06–3.10 (m, 1H), 3.19 (d, J = 18.2 Hz, 1H), 3.26 (d, J = 19.6 Hz, 1H), 3.28 (s, 3H), 3.33 (s, 3H), 3.04–3.44 (m, 1H), 3.49–3.59 (m, 3H), 4.01–4.15 (m 4H), 4.76 (s, 2H) 6.67 (s, 1H), 7.34 (t, J = 7.0 Hz, 1H), 7.40 (t, J = 7.0 Hz, 2H), 7.47 (d, J = 7.0 Hz, 2H), 7.71 (s, 1H); ^{13}C NMR (176 MHz, $CDCl_3$): δ 14.0, 16.2, 16.3, 18.9, 27.8, 29.5, 31.30, 31.32, 33.2, 37.0, 39.5, 45.9, 46.6, 47.9, 51.0, 55.6, 58.7, 69.1, 73.8, 74.8, 123.4, 127.6, 127.9, 128.5, 129.8, 131.5, 133.4, 135.5, 137.5, 141.0, 142.0, 143.8, 145.2, 154.0, 154.5, 171.0; HRMS (ESI) *m/z* calc'd. for $C_{39}H_{55}N_3O_{10}S_3$ $[M+H]^+$ 822.3122, found 822.3125.

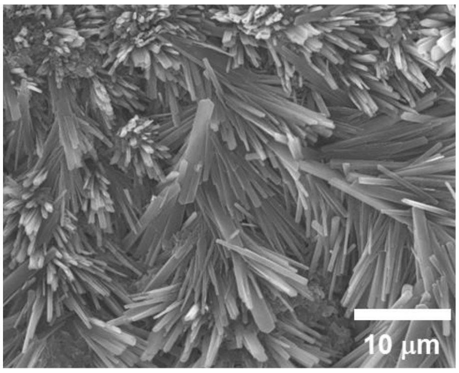

Supplementary Figure 3. SEM image of brinzolamide microstructures in Azopt.

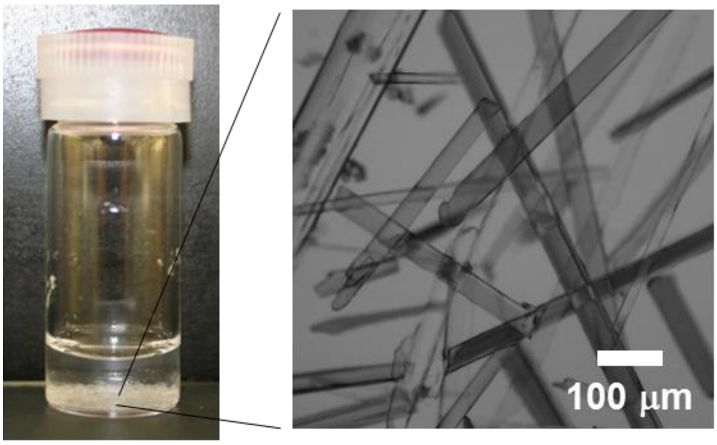

Supplementary Figure 4. Photograph of brinzolamide aqueous dispersion prepared by the reprecipitation method and microscopic image of resulting brinzolamide micro-sized fibres.

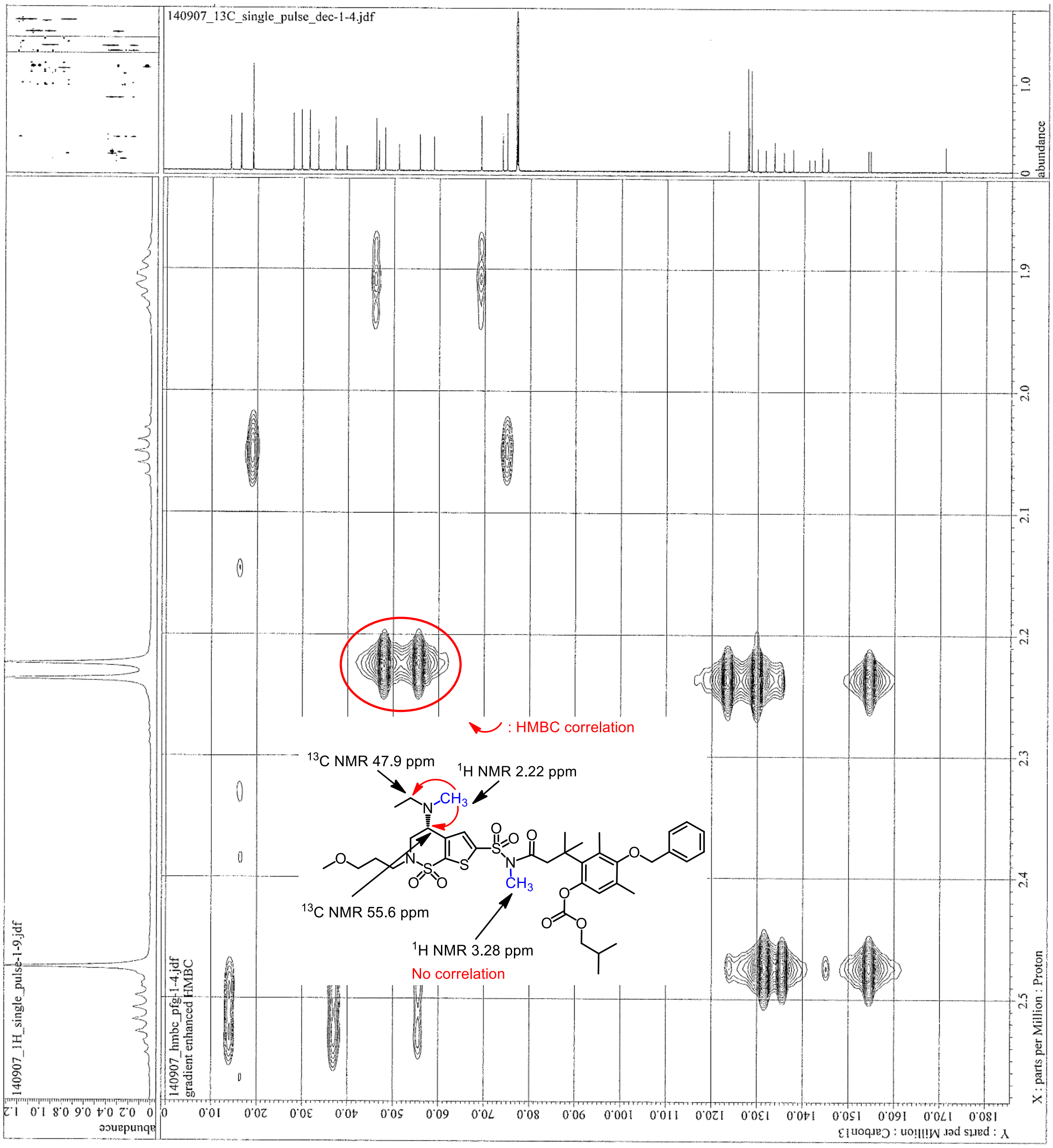

Supplementary Figure 5. Magnified image of the HMBC experiment of the dimethylated TML prodrug.

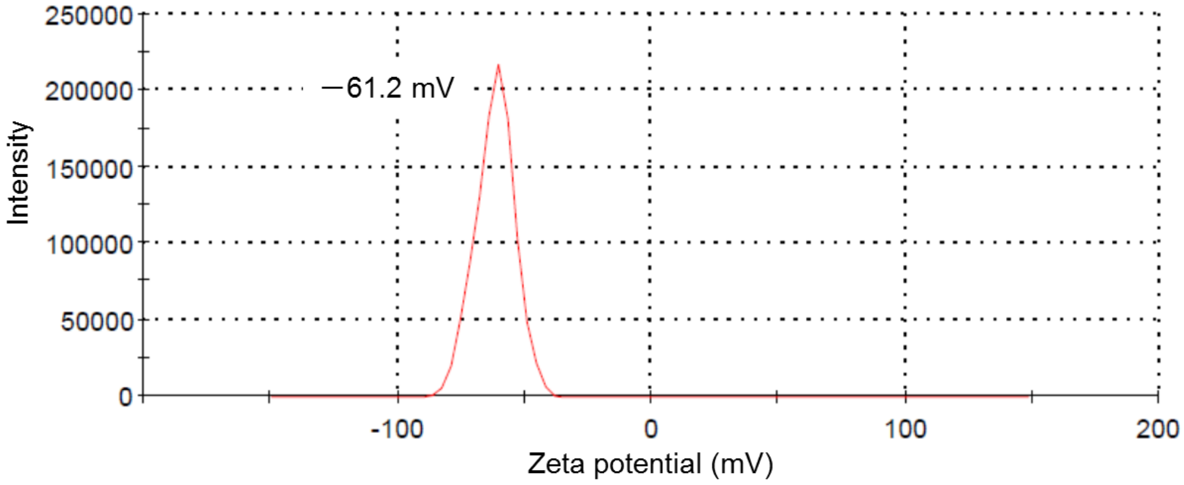

Supplementary Figure 6. Zeta potential of TML prodrug nanoparticles.

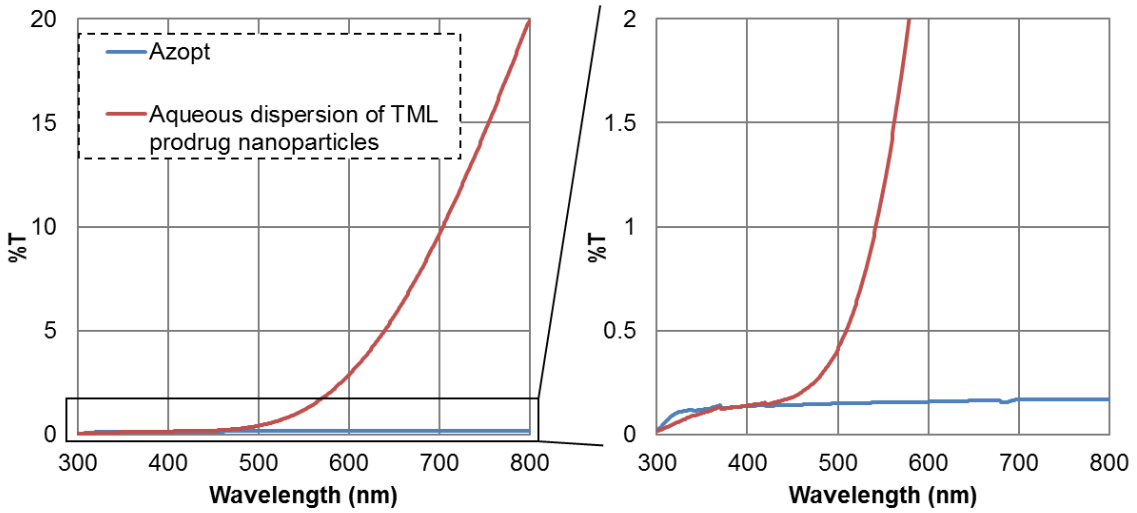

Supplementary Figure 7. Transmittance of Azopt and aqueous dispersion of TML prodrug nanoparticles.

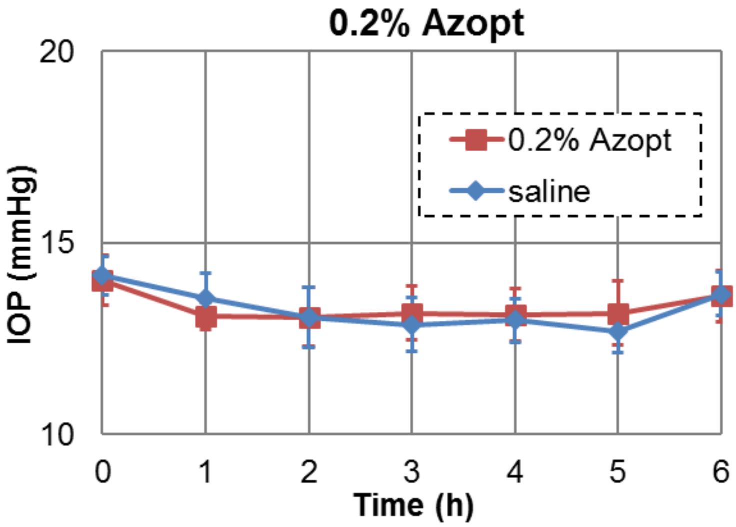

Supplementary Figure 8. Ocular hypotensive effect of 0.2% Azopt.

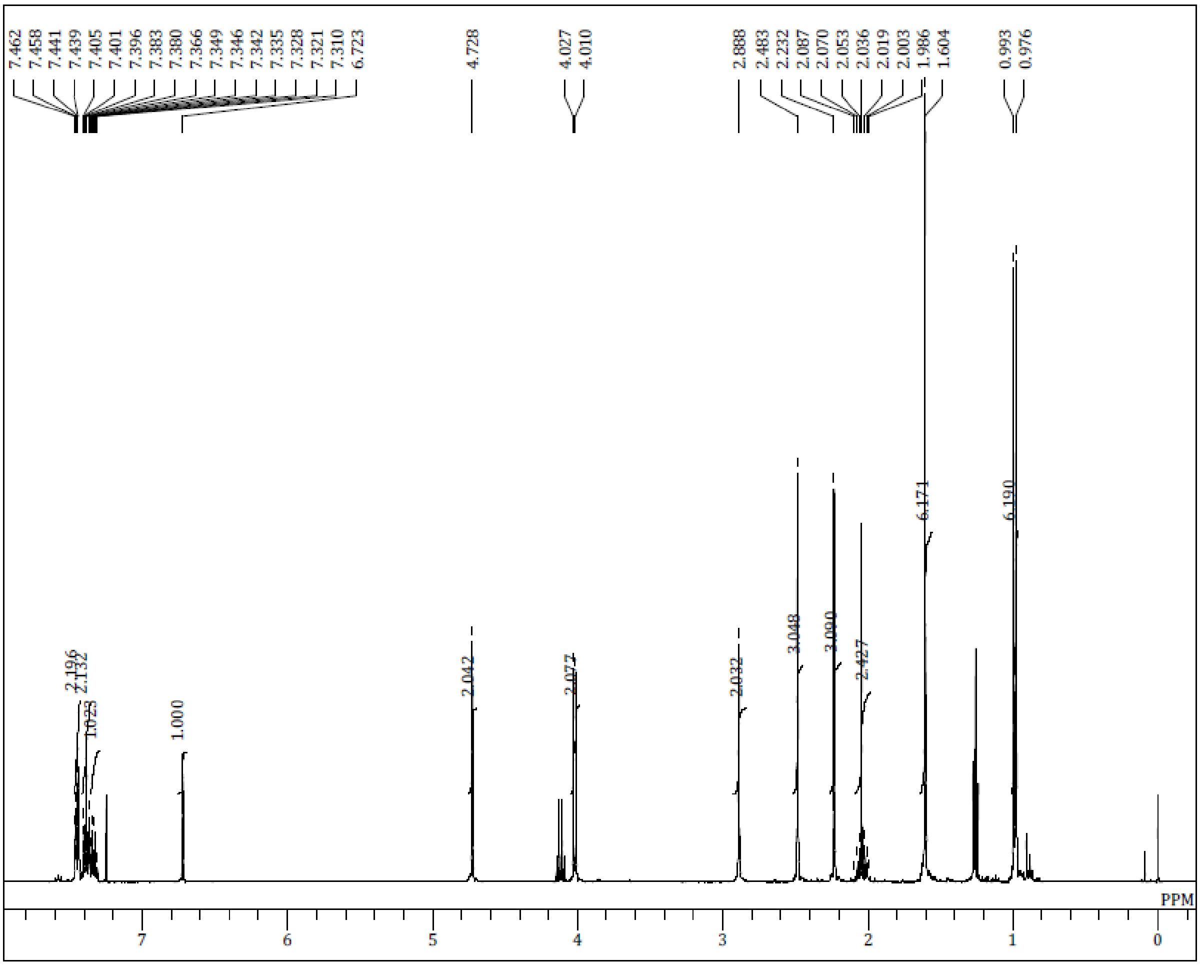

Supplementary Figure 9. ^{1}H NMR spectra of **7**.

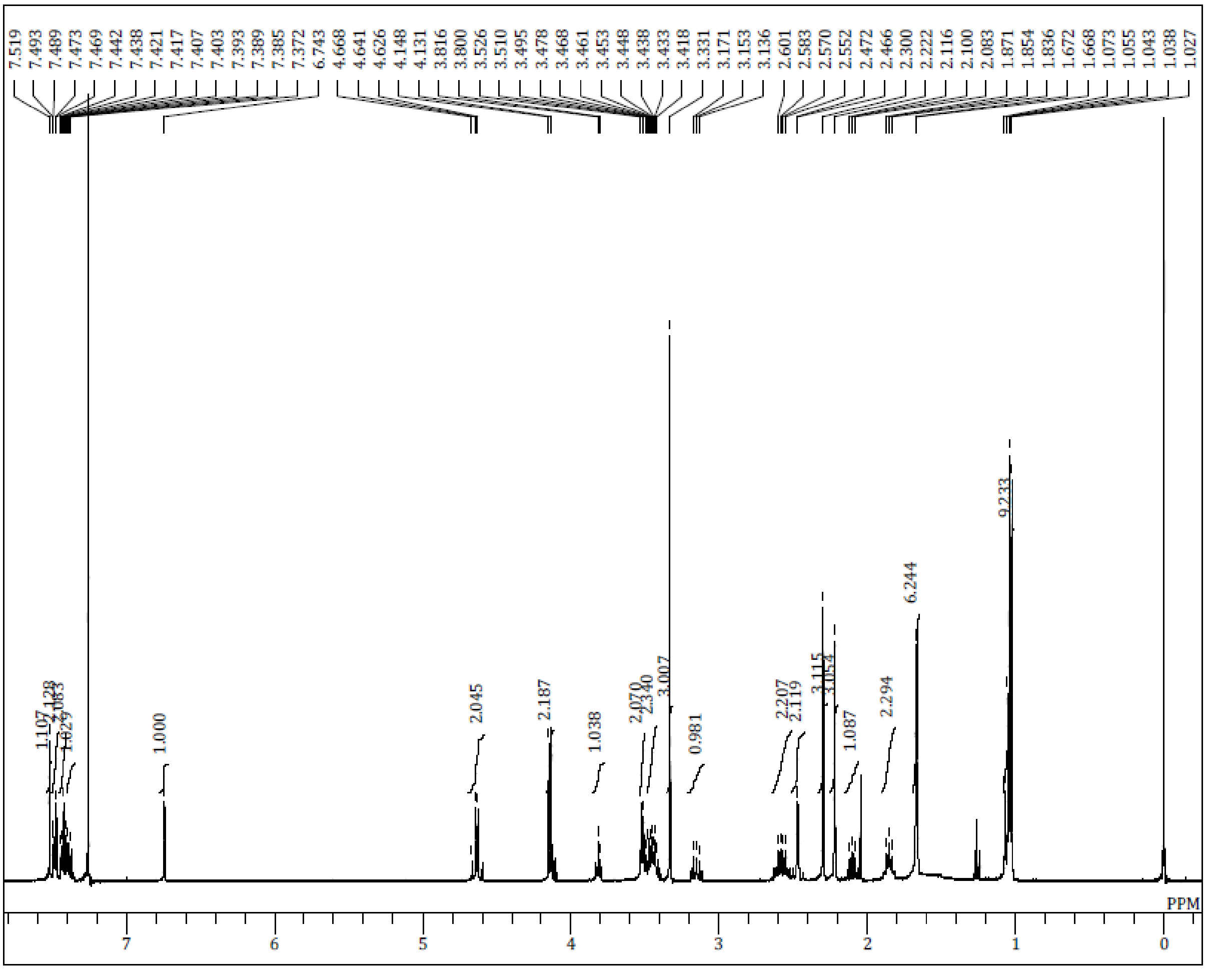

Supplementary Figure 10. ^{1}H NMR spectra of the TML prodrug.

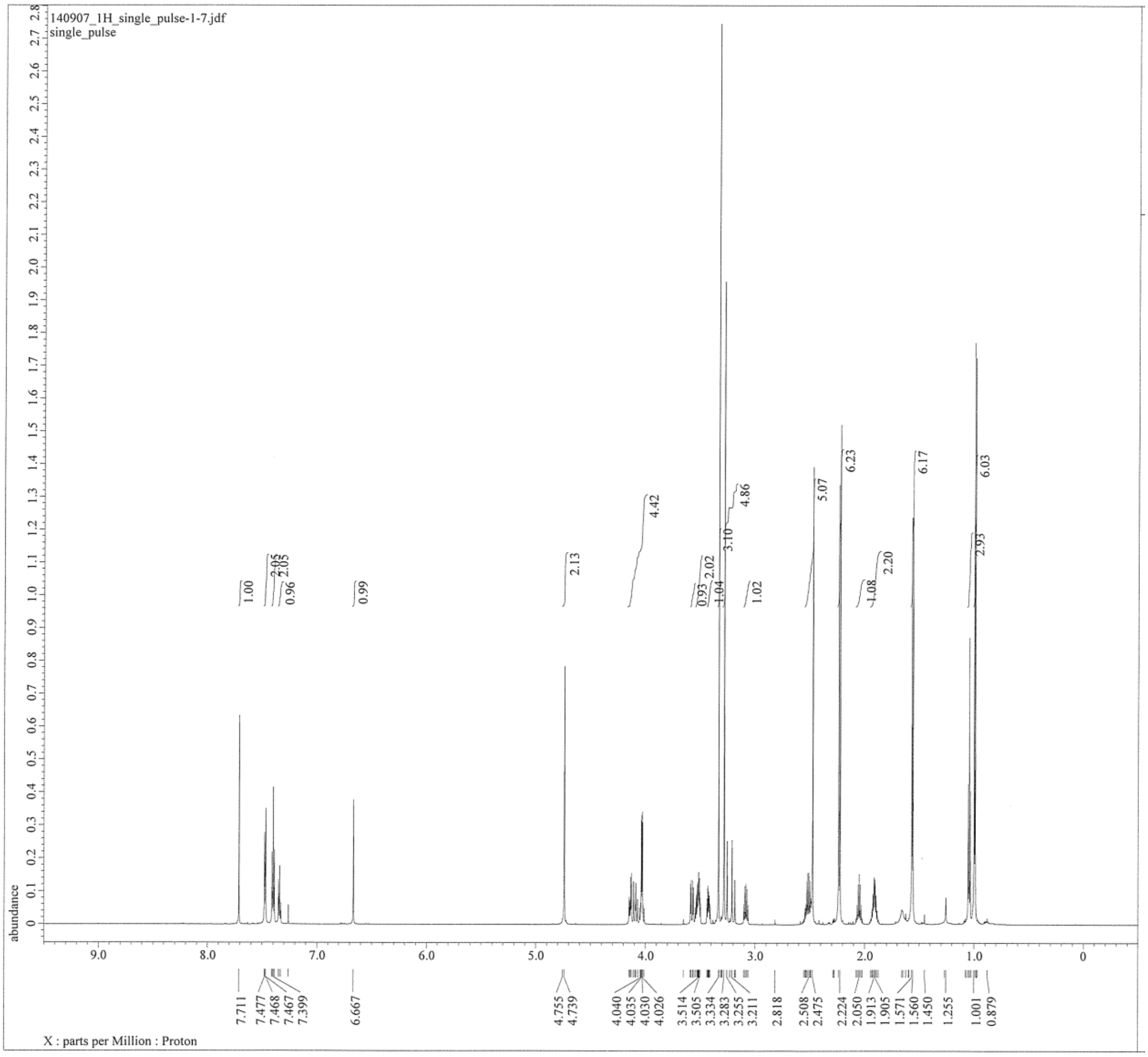

Supplementary Figure 11. ^{1}H NMR spectra of the dimethylated TML prodrug.

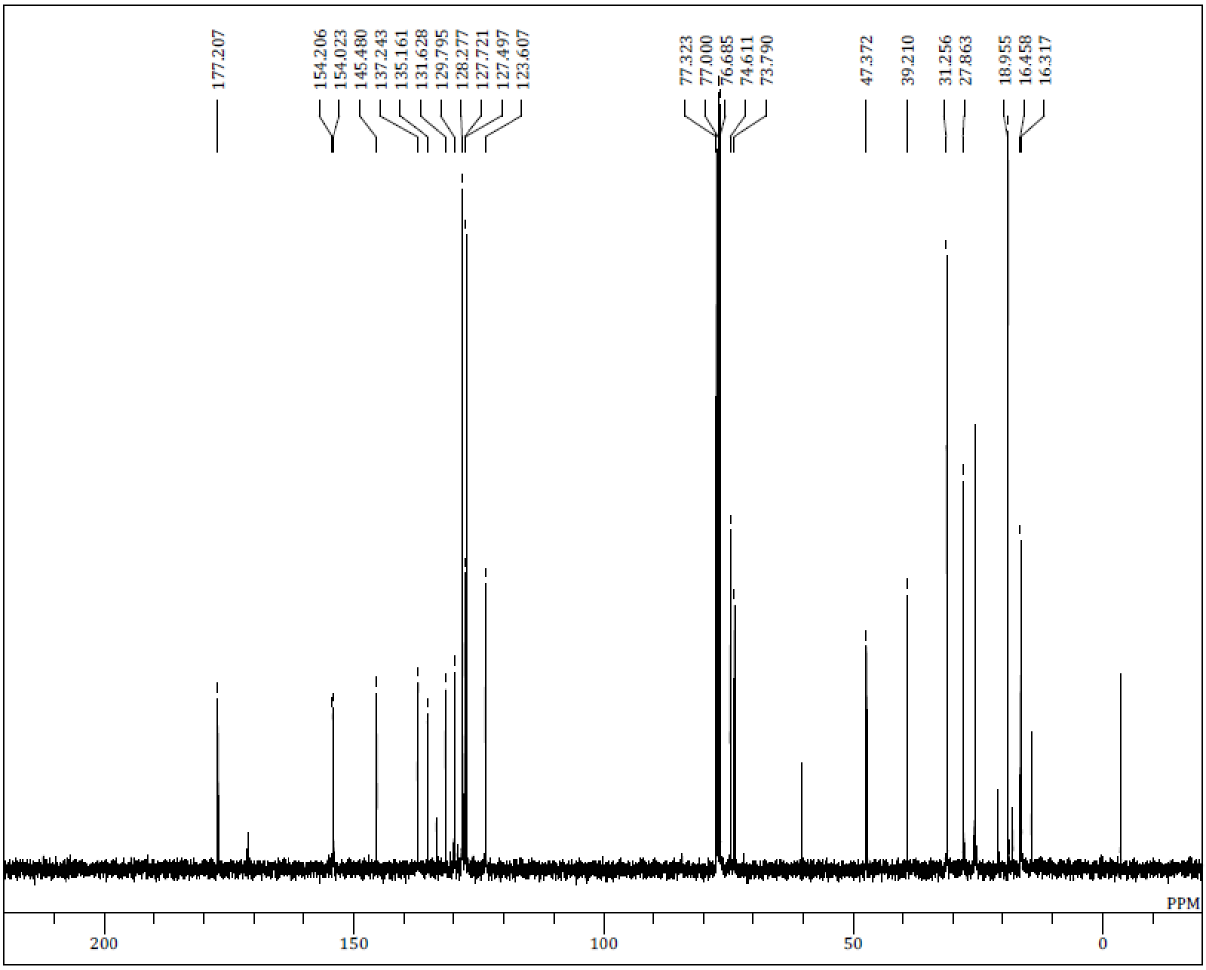

Supplementary Figure 12. ^{13}C NMR spectra of **7**.

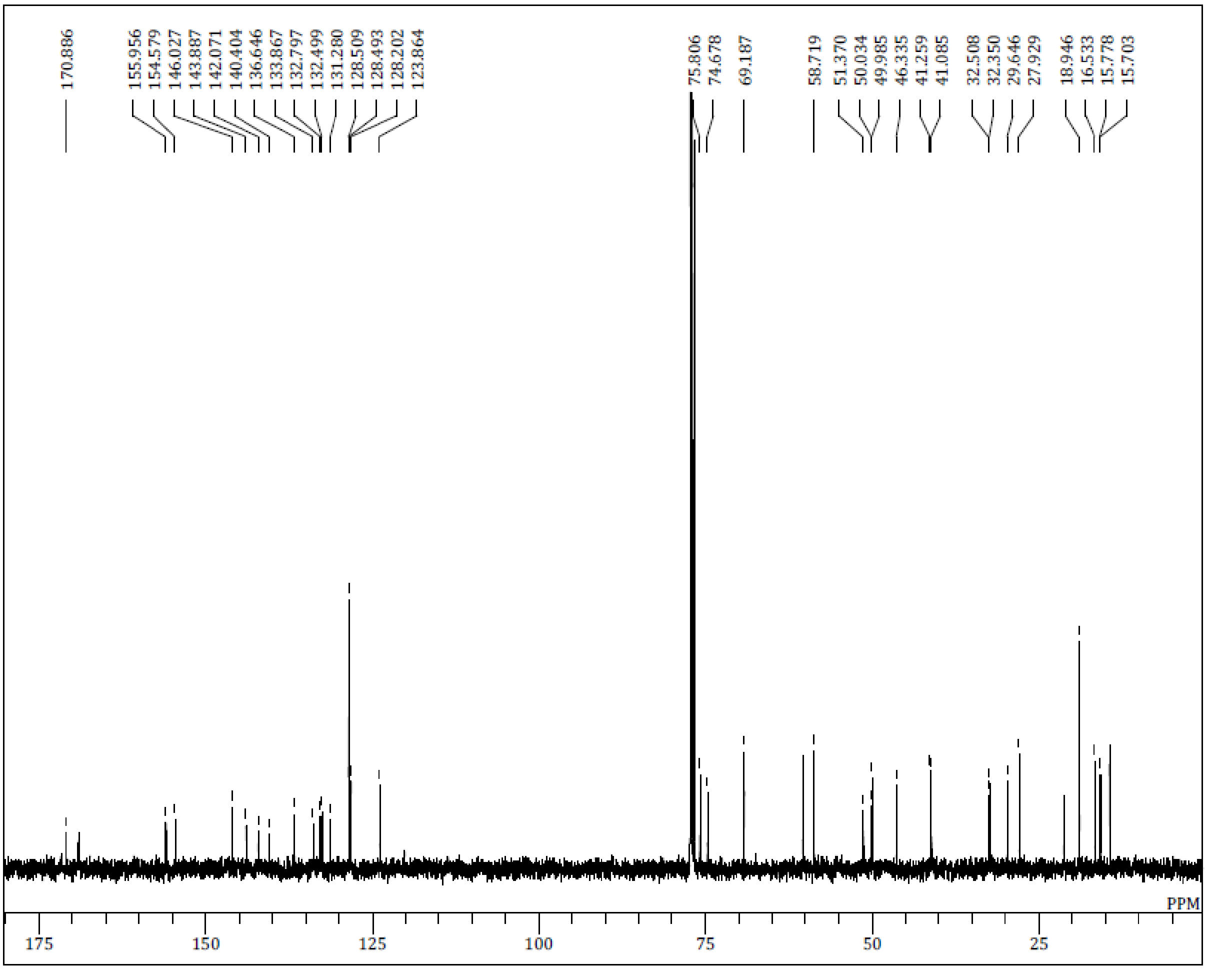

Supplementary Figure 13. ^{13}C NMR spectra of the TML prodrug.

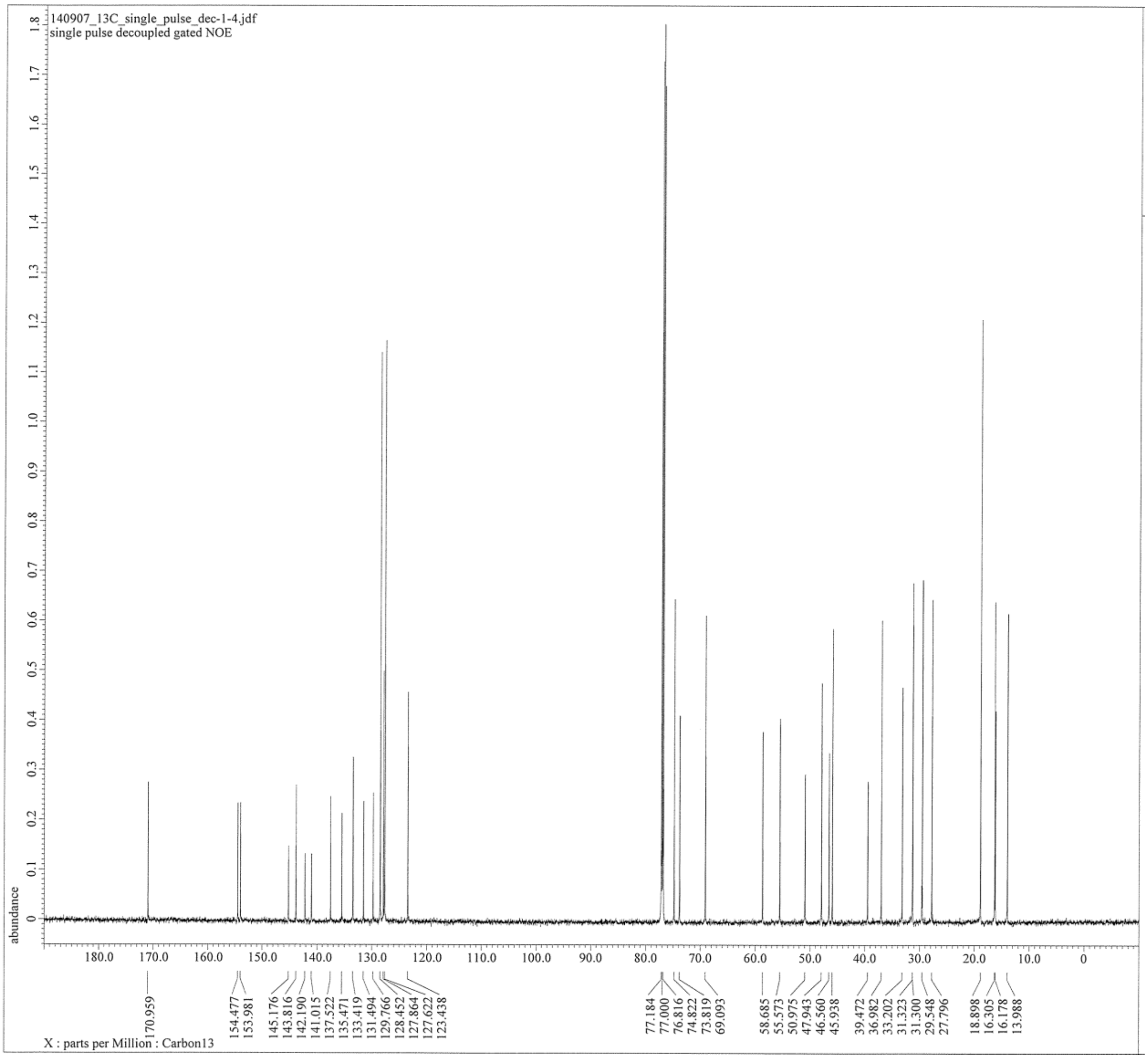

Supplementary Figure 14. ^{13}C NMR spectra of the dimethylated TML prodrug.

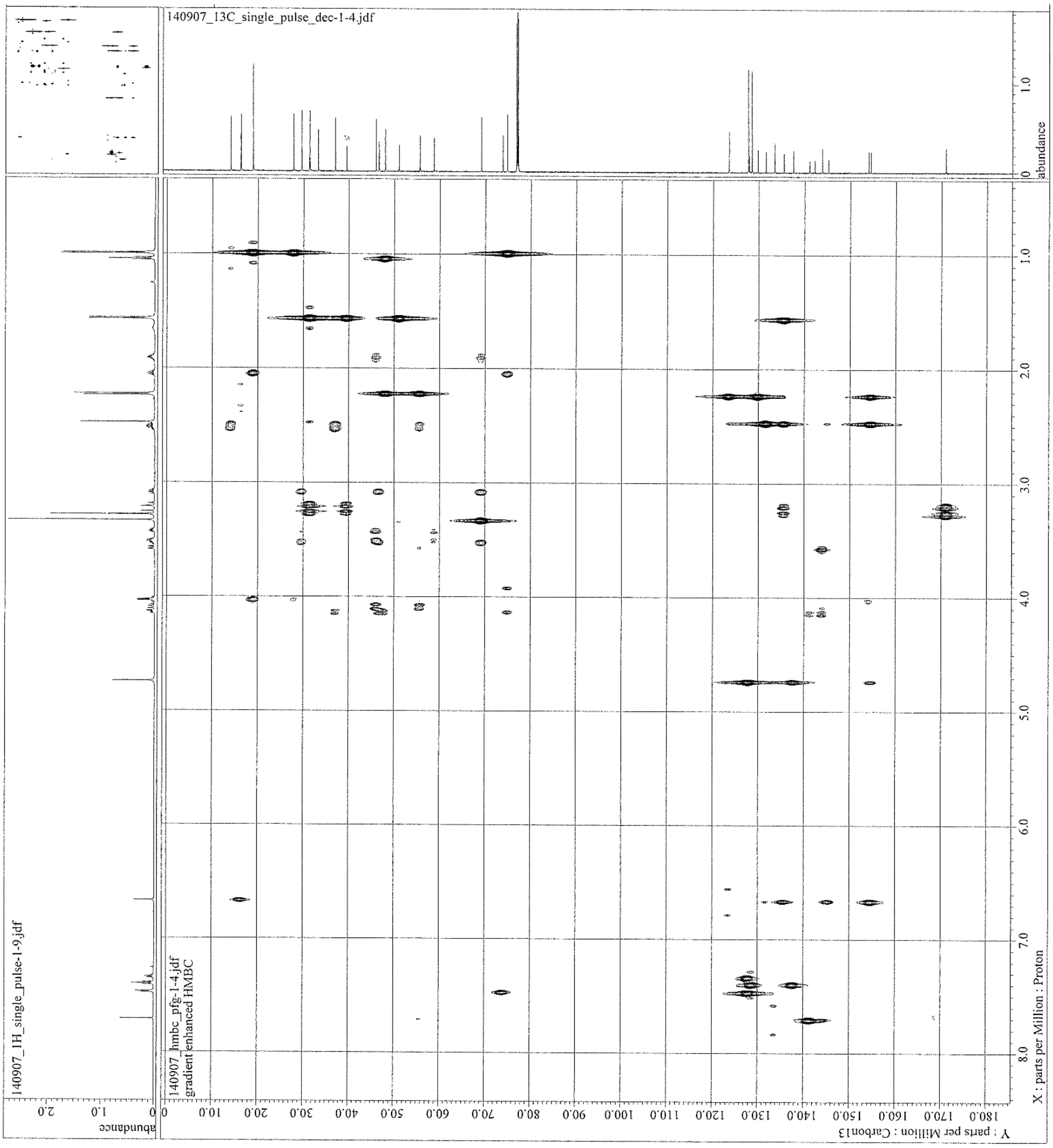

Supplementary Figure 15. HMBC experiment of the dimethylated TML prodrug.

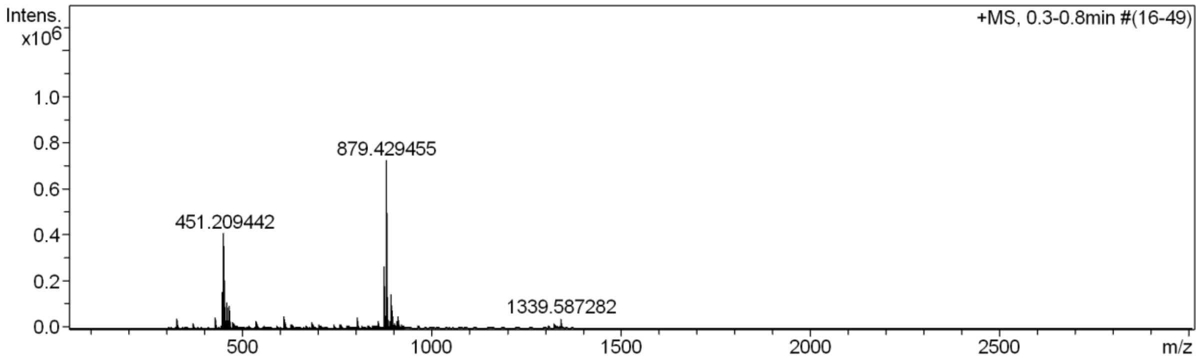

Supplementary Figure 16. HRMS spectra of **7**.

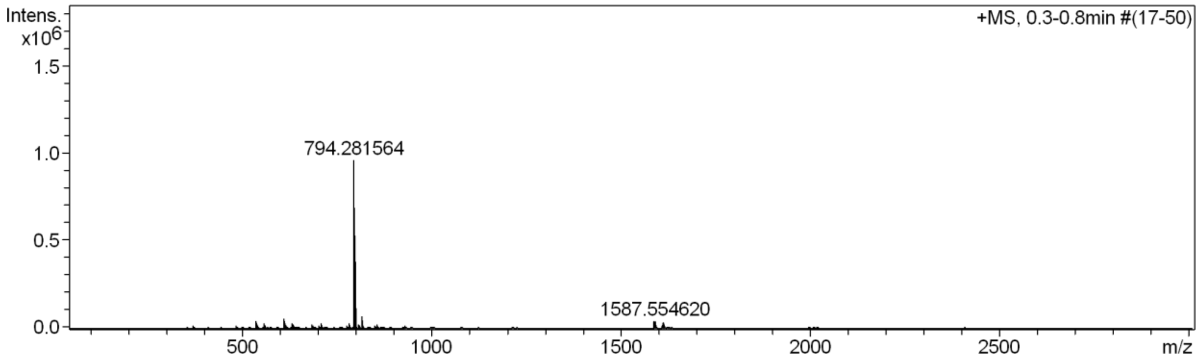

Supplementary Figure 17. HRMS spectra of the TML prodrug.

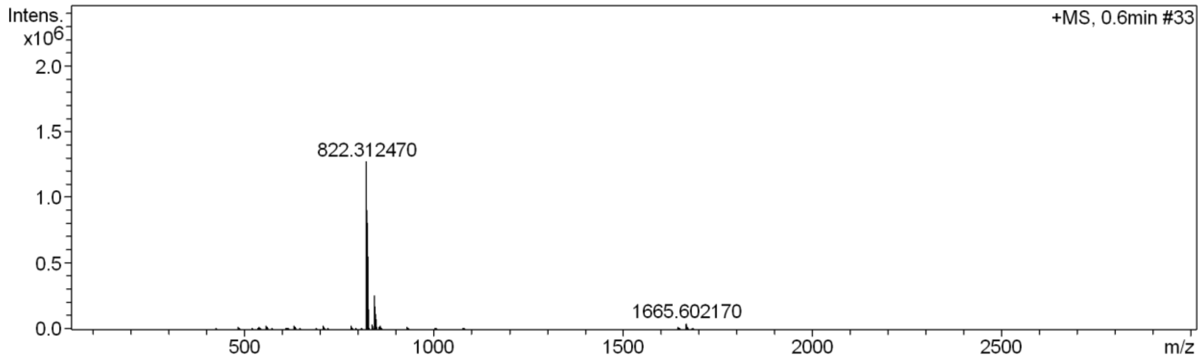

Supplementary Figure 18. HRMS spectra of the dimethylated TML prodrug.

References

1. Shan, D. X., Zheng, A. L., Ballard, C. E., Wang, W., Borchardt, R. T. & Wang, B. H. A facilitated cyclic ether formation and its potential application in solid-phase peptide and organic synthesis. *Chem. Pharm. Bull.* **48**, 238-244 (2000).
